# Evaluation of machine learning pipeline for blood culture outcome prediction on prospectively collected emergency department data

**DOI:** 10.1099/jmm.0.002191

**Published:** 2026-07-29

**Keywords:** blood cultures, bloodstream infection, machine learning

## Abstract

**Introduction.** Bloodstream infections (BSIs) are particularly problematic in the emergency department (ED) of hospitals, where patients often present with undiagnosed illness, one cause of which may be undetected BSI. Identifying whether a patient needs a blood culture (BC) performed is one component of this challenge with implications for diagnostic efficiency and avoidance of unnecessary resource expenditure.

**Gap statement.** In Western Australia, there has been no previous study investigating methods to predict BC outcome in an ED patient cohort.

**Aim.** This article assesses the feasibility of previously developed machine learning (ML) models for BC outcome prediction using a prospectively collected ED patient dataset.

**Methodology.** An ML pipeline containing models previously trained using complete blood count (CBC), white blood cell differential (DIFF) and cell population data (CPD) generated by Sysmex XN-2000 haematology analysers was further evaluated using prospectively collected data containing patient sample results from the ED at Sir Charles Gairdner Hospital (SCGH), Perth, Western Australia. Blood samples used to produce CBC, DIFF and CPD were obtained at the same time as BC samples.

**Results.** There were 59 samples from 58 unique patients. Forty-nine of those samples were associated with negative BC results and 10 with positive BC results. We evaluated previously developed XGBoost (XG) and random forest (RF) ML models for positive BC outcome prediction. The RF and XG models obtained mean area under the receiver operating characteristic curve scores of 0.865 (95% CI, 0.763–0.947) and 0.833 (95% CI, 0.683–0.953) with the ED dataset.

**Conclusion.** The results presented in this study provide a foundation for further validation and shadow deployment of BC outcome prediction models in clinical settings and support future planning of clinical trials.

**Impact Statement**

This study is the first in Western Australia to examine how machine learning can help predict bloodstream infection in emergency department patients. By validating previously developed prediction models with a new set of patient data, we demonstrate that these tools can flag which patients might need blood cultures, potentially improving diagnostic accuracy and resource use. This adds to existing literature by applying the models to a patient cohort in which patients arrive with complex, undiagnosed conditions. The approach will be of interest to emergency department clinicians and to laboratory medicine and healthcare administrators seeking to improve efficiency and patient care. While the findings come from a relatively small sample, they represent an important step forward. The encouraging results support larger-scale evaluations and provide the foundation for clinical trials, aiming ultimately to refine and implement these models in everyday practice.

## Introduction

Bloodstream infections (BSIs) are a heterogeneous group of infectious diseases characterized by the presence of pathogenic micro-organisms in the blood. BSIs can result in significant inflammatory responses characterized by changes in clinical, laboratory and haemodynamic parameters [1]. When left untreated, a BSI can result in life-threatening organ dysfunction due to a dysregulated immune response to the BSI known as sepsis [2]. Individuals with chronic illnesses, those at the extremes of age and individuals that are immune compromised are at increased risk of developing BSIs [3]. BSIs can occur in the community, in hospitals and other healthcare environments and can be a complication of other infectious diseases including pneumonia, urinary tract infections, endocarditis, catheter-associated infections and surgical site infections [4]. In some cases, an original source of the BSI cannot be determined and is referred to as a primary BSI. Blood culture (BC) testing is used to identify the presence of bacteria or fungi in the blood. A BSI is identified by the positivity (micro-organism growth) in one or more BCs and is considered the current standard for the diagnosis of BSIs. The BC test is one stage of a comprehensive process for the identification of BSIs in patients. This includes the assessment and identification of patients with a suspected BSI, BC request by clinicians, additional laboratory test requests, collection of blood from patients, transport of samples, sample incubation, initial growth detection, Gram staining, organism identification and antimicrobial susceptibility testing [5–7]. However, the BC process has limits. Most notably, it is widely overused and has a low positive yield [8, 9]. The downstream effects of this include increased length of stay in hospital, additional patient testing, overuse of antimicrobials and significant increases in cost and resource expenditure for laboratories and hospitals, especially in the case of false positive results [6, 9–13]. This highlights the importance of improving the BSI care pathway. In the emergency department (ED), opportunities exist to make significant improvements in BC use, as it is often the first point of contact between patients and the hospital setting. Machine learning (ML) can be regarded as a set of methods that identify patterns from data [14]. ML has recently been applied to a wide range of areas relating to infectious diseases such as sepsis [15], antimicrobial resistance [16], pneumonia [17] and viral outbreak detection [18].

In our previous work, we developed an ML pipeline for BC outcome prediction which achieved promising results using models trained with complete blood count (CBC), white blood cell (WBC) differential (DIFF) and cell population data (CPD) [19]. The ML pipeline was originally developed to use CBC, DIFF and CPD from Sysmex XN-2000 haematology analysers (Sysmex, Kobe, Japan). While CBC and DIFF results are routinely reported in laboratories, CPD data are used only for research. Retrospective data from 1 January 2018 to 31 May 2020 were used for model training. CBC, DIFF and CPD results were linked with matching BC outcomes from the laboratory information system (LIS), including only adult samples where the sample identification number for CBC, DIFF and CPD test results matched the sample identification number of the BC. The training dataset contained 10,965 samples, of which 831 (7.6%) were blood-culture-positive. In this study, we further evaluated this pipeline using a prospectively collected ED dataset.

## Methods

### Data collection

Blood samples were collected as part of a prospective observational study with convenience sampling from patients in the ED at Sir Charles Gairdner Hospital (SCGH), Perth, Western Australia. Data was obtained between August 2020 and November 2021. Blood samples were collected from adult patients above 18 years of age who were able to provide informed consent to participate and required BCs as part of their clinical assessment for suspicion of BSI. Patients were recruited by medical and nursing staff after patients were admitted to the ED at SCGH during the time period. Patients were not recruited whilst COVID-19 restrictions were in place due to clinical research activities being regarded as non-essential in Western Australia during the heights of COVID-19 restrictions. The research was conducted in accordance with the National Health and Medical Research Council (NHMRC) ethics guidelines and Good Clinical Practice guidelines.

#### ML pipeline

**Fig. 1.**
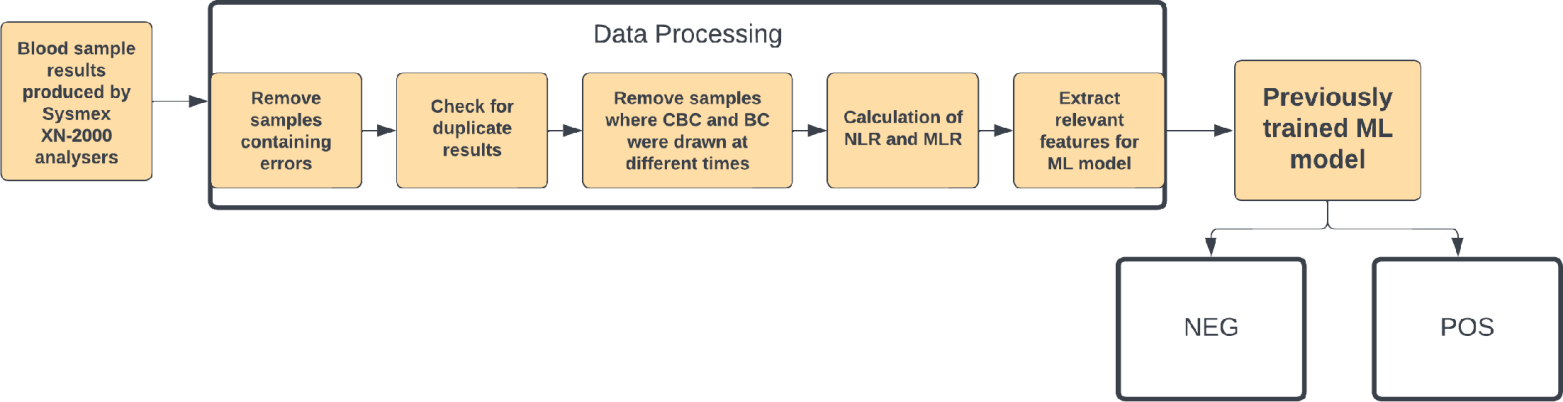
Evaluation pipeline for BC outcome prediction ML models. The ML models predict either a negative BC (NEG) or positive BC (POS) result using CBC, DIFF and CPD. The models also use neutrophil–lymphocyte ratio (NLR) and monocyte–lymphocyte ratio (MLR) derived from CBC data.

The two models evaluated in this study were trained in our previous work [19]. The XGBoost (XG) model was trained using CBC, DIFF and CPD features with boruta feature selection (BFS) and 1.5 class weights in favour of the positive BC result; and the random forest (RF) model was trained using CBC and DIFF features, BFS and balanced class weights. The features used in each of these models and descriptions for these features are presented in Table 1. No further model re-training was performed after the initial development of these models. The ML model evaluation pipeline is shown in Fig. 1. ML models were evaluated based on their ability to correctly classify positive BC (POS) or negative BC (NEG) outcomes. To evaluate the performance of the models in the ML pipeline, the area under the receiver operating characteristic curve (auROC), sensitivity, specificity, negative predictive value (NPV) and positive predictive value (PPV) were used. Descriptions and equations for these metrics are shown in Table S1 (available in the online Supplementary Material). The ML pipeline used in this study was developed with the Python programming language (version 3.12).

**Table 1.** Descriptions for each of the features required for the XG and RF models. n/a indicates the feature is not used in the model

XG model	RF model	Description
[MO-WZ]	n/a	Width of dispersion of monocytes size
NEUT#(10^9^ /L)	NEUT#(10^9^ /L)	Absolute neutrophil count
[NE-SFL(ch)]	n/a	Neutrophil fluorescence intensity
LYMPH#(10^9^ /L)	LYMPH#(10^9^ /L)	Absolute lymphocyte count
IP SUS(WBC)Left Shift	n/a	Suspected presence of immature neutrophils
[MO-WY]	n/a	Width of dispersion of monocytes fluorescence
RDW-CV(%)	RDW-CV(%)	Red blood cell distribution width
[NE-FSC(ch)]	n/a	Neutrophils forward scatter
LYMPH%(%)	LYMPH%(%)	Lymphocyte differential relative percentage
MONO#(10^9^ /L)	MONO#(10^9^ /L)	Absolute monocyte count
MONO%(%)	MONO%(%)	Monocyte differential relative percentage
NLR	NLR	Neutrophil-lymphocyte ratio
EO%(%)	EO%(%)	Eosinophil differential relative percentage
BASO%(%)	BASO%(%)	Basophil differential relative percentage
[NE-WY]	n/a	Width of dispersion of neutrophils fluoresence
NEUT%(%)	NEUT%(%)	Neutrophil differential relative percentage
n/a	HGB(g/L)	Haemoglobin
n/a	RBC(10^12^ /L)	Red blood cell count
n/a	BASO#(10^9^ /L)	Absolute basophil count
n/a	PLT(10^9^ /L)	Platelet count
n/a	WBC(10^9^ /L)	White blood cell count
n/a	EO#(10^9^ /L)	Absolute eosinophil count
n/a	MLR	Monocyte-lymphocyte ratio

### Dataset processing

Blood samples collected from patients were used to perform CBC, DIFF, CPD, BC and C-reactive protein (CRP) tests. Samples were collected at the same time, sharing sample identification numbers in the LIS. The CBC, DIFF and CPD data for each patient was obtained directly from the archived datasets downloaded directly from the Sysmex XN-2000 haematology analysers (Sysmex, Kobe, Japan). Additional features required including neutrophil–lymphocyte ratio (NLR) and monocyte–lymphocyte ratio (MLR) were calculated based on the DIFF results. Data relating to BCs, CRP and patient demographics including age and sex were obtained from the LIS. Ninety-seven patients with BCs taken were initially present in the dataset. Patient samples were included if (1) CBC and BC samples were drawn at the same time and (2) the CBC test was an initial test. The dataset was also checked for duplicates. Missing data was not imputed, and samples that had missing CBC, DIFF and CPD values were removed from the dataset. Samples were removed if errors were present during the haematology analysis, identified automatically by the Sysmex XN-2000 analysers. After performing these checks, 59 remained in the final dataset. The BC test results, which represented the ground truth labels for classification, were considered positive if there was growth of a microorganism in the BC. The positive results were confirmed as clinically significant after reviewing the discharge summaries of the patients involved in the study.

### Exploratory data analysis

Patient ages ranged from 20 to 93 years, with 49 NEG results and 10 POS results. For those with NEG results, the median age was 64 with an interquartile range (IQR) of 30, with 29 males and 20 females. For the 10 samples with a POS result, the median age was 65.5 with an IQR of 27.25, with 5 males and 5 females. The micro-organisms isolated from the POS results include *Escherichia coli* (3), *Klebsiella pneumoniae* (2), *Streptococcus pyogenes* (1), *Staphylococcus aureus* (1), *Staphylococcus hominis* (1), *Streptococcus bovis* (1) and *Streptococcus dysgalactiae* (1). For the NEG and POS results, median and IQR values are shown in Table 2 for all relevant ML features, and CRP, which was available for 56 of the 59 patients.

**Table 2.** Median and IQRs for relevant features in the dataset shown for positive BC (POS) and negative BC (NEG) results. [MO-WZ] (Width of dispersion of monocytes size); NEUT#(10^9^/L) (absolute neutrophil count); [NE-SFL(ch)] (neutrophil fluorescence intensity); LYMPH#(10^9^/L) (absolute lymphocyte count); IP SUS(WBC)Left (shift suspected presence of immature neutrophils); [MO-WY] (width of dispersion of monocytes fluorescence); RDW-CV(%) (red blood cell distribution width); [NE-FSC(ch)] (neutrophils forward scatter); LYMPH%(%) (lymphocyte differential relative percentage); MONO#(10^9^/L) (absolute monocyte count); MONO%(%) (monocyte differential relative percentage); NLR (neutrophil–lymphocyte ratio); EO%(%) (eosinophil differential relative percentage); BASO%(%) (basophil differential relative percentage); [NE-WY] (width of dispersion of neutrophils fluorescence); NEUT%(%) (neutrophil differential relative percentage); HGB(g/L) (haemoglobin); RBC(10^12^/L) (red blood cell count); BASO#(10^9^/L) (absolute basophil count); PLT(10^9^/L) (platelet count); WBC(10^9^/L) (white blood cell count); EO#(10^9^ /L) (absolute eosinophil count); MLR (monocyte–lymphocyte ratio); CRP (C-reactive protein)

Feature	NEG (*n*=49)	POS (*n*=10)
CBC, DIFF and CPD features, median (IQR)		
[MO-WZ]	529.0 (101.0)	526.5 (124.25)
NEUT#(10^9^/L)	9.1 (8.74)	12.38 (2.41)
[NE-SFL(ch)]	49.5 (5.0)	49.05 (7.30)
LYMPH#(10^9^/L)	0.94 (0.76)	0.525 (0.345)
[MO-WY]	736.0 (126.0)	777.5 (204.5)
RDW-CV(%)	13.6 (2.0)	15.1 (2.53)
[NE-FSC(ch)]	89.5 (5.7)	88.3 (5.58)
LYMPH%(%)	8.0 (8.9)	3.9 (0.675)
MONO#(10^9^/L)	0.89 (0.82)	0.65 (0.42)
MONO%(%)	7.2 (5.6)	4.0 (3.63)
NLR	11.06 (9.75)	23.09 (4.72)
EO%(%)	0.2 (0.8)	0.0 (0.1)
BASO%(%)	0.2 (0.2)	0.2 (0.2)
[NE-WY]	664.0 (84.0)	743 (164.5)
NEUT%(%)	82.7 (17.8)	91.25 (5.03)
HGB(g/L)	128.0 (26.0)	125 (18.5)
RBC(10^12^/L)	4.23 (0.94)	4.19 (1.02)
BASO#(10^9^/L)	0.03 (0.03)	0.03 (0.04)
PLT(10^9^/L)	206.0 (155.0)	177 (151.25)
WBC(10^9^/L)	10.13 (9.05)	13.64 (2.41)
EO#(10^9^/L)	0.02 (0.12)	0.0 (0.01)
MLR	0.96 (1.18)	1.07 (0.87)
CRP	91.5 (129.25)	172.5 (217.5)

### Haematology features

The ML models evaluated in this study use haematological data produced by Sysmex XN-2000 module analysers, encompassing the CBC, DIFF and CPD features. A CBC, a common laboratory test, evaluates blood samples for cellular components such as WBC, platelets and red blood cells (RBC). Beyond CBC, DIFF testing is often performed to assess the proportion of WBC types, including neutrophils (NEUT), lymphocytes (LYMPH), monocytes (MONO), basophils (BASO) and eosinophils (EO). The DIFF data can be used to calculate ratios like NLR and MLR. Equations for NLR and MLR are shown in Fig. S9. CPD features, derived from the fluorescent flow cytometry technology used in Sysmex analysers, quantify side scatter light (SSC), forward scatter light (FSC) and fluorescent light intensity (SFL). These metrics are visually represented in a scattergram, aligning SSC with cellular granularity, FSC with cell volume and shape and SFL with nucleic acid and protein levels in cells [20, 21]. Additionally, the Sysmex XN-2000 generates interpretive programme messages (IP flags) after CBC tests, alerting to potential haematological issues, with specific flags for WBC, RBC and PLT anomalies [22].

### C-reactive protein

CRP is a protein that is made in the liver and released into the bloodstream. The levels of CRP in plasma may rise in response to acute inflammatory stimulus [23]. The stimulus for secretion of CRP by the liver is the pro-inflammatory cytokine, 1L-6 [24]. A normal CRP level can be considered as less than 0.3 mg dl^−1^. More than 10.0 mg dl^−1^ may be the result of acute bacterial infections, viral infections or trauma, while levels higher than 50 mg dl^−1^ are associated with bacterial infections [25].

### Quick sequential organ failure assessment and systemic inflammatory response syndrome

The quick sequential organ failure assessment (qSOFA) score [2] and the systemic inflammatory response syndrome (SIRS) criteria [26] are two scoring systems often used to identify patients at risk of sepsis and/or a BSI. A patient is considered to meet the SIRS criteria if they meet two or more of the following clinical elements: (1) temperature >38 degrees Celsius or temperature <36 degrees Celsius; (2) heart rate greater than 90 per minute; (3) respiratory rate >20 per minute or partial pressure of arterial carbon dioxide (PaCO_2_) <32 mm Hg; and (4) WBC>12,000 /mm^3^, 4,000 /mm^3^ or >10% immature bands. For the qSOFA score, a patient who meets at least two of the following criteria should have infection considered: (1) altered mental status based on a Glasgow Coma Scale score <15, (2) respiratory rate ≥22 per minute and (3) systolic blood pressure ≤100. The qSOFA scores and clinical elements required to determine the SIRS criteria were both available for patients in this study.

## Results

### Model performance evaluation

To evaluate the predictive performance of the ML models, both bootstrap resampling and direct evaluation were employed. When applying bootstrapping, the XG model achieved a mean auROC of 0.833 [95% confidence interval (CI), 0.683–0.953], indicating good discriminative ability. Without bootstrapping, at a classification threshold of 0.5, the XG model demonstrated an NPV of 0.926, PPV of 0.250, sensitivity of 0.800 and specificity of 0.510.

The RF model performed slightly better overall, achieving a mean auROC of 0.865 (95% CI, 0.763–0.947) under bootstrapping. When evaluated without resampling at the 0.5 threshold, the RF model produced perfect sensitivity (1.0) and NPV (1.0), alongside a PPV of 0.323 and a specificity of 0.571, suggesting that the RF model was highly sensitive to positive cases while maintaining moderate specificity.

**Fig. 2.**
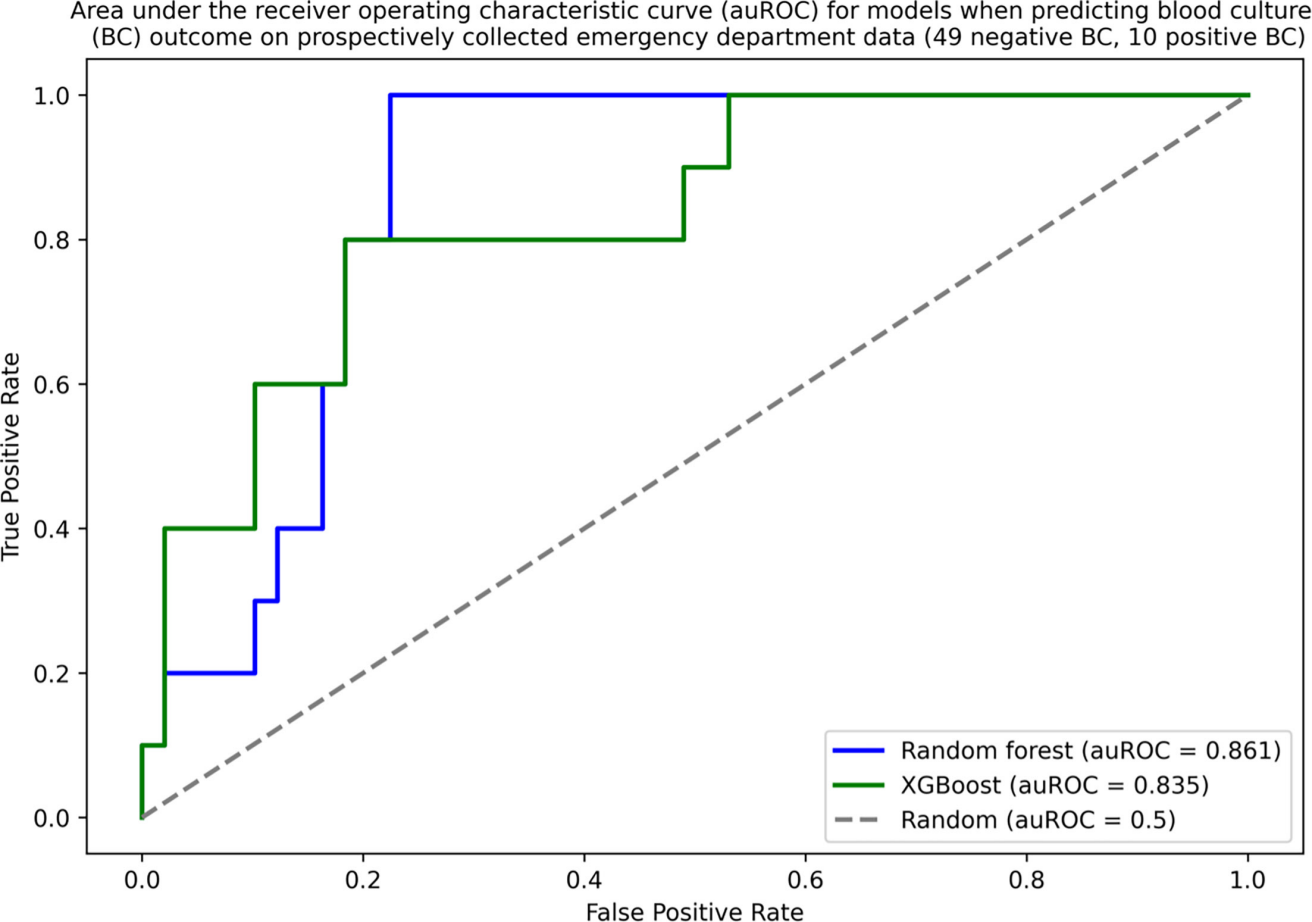
auROC for the RF and XG models for prediction of BC outcomes on the entire dataset (49 negative BCs, 10 positive BCs).

A detailed examination of model performance across varying decision thresholds (0.1–0.9) is provided in the Figs S1–S4, which include the corresponding confusion matrices for both uncalibrated and calibrated RF and XG models. The ROC curves and associated auROC scores for the models evaluated on the full dataset are presented in Fig. 2, providing a visual comparison of their discriminative performance.

**Fig. 3.**
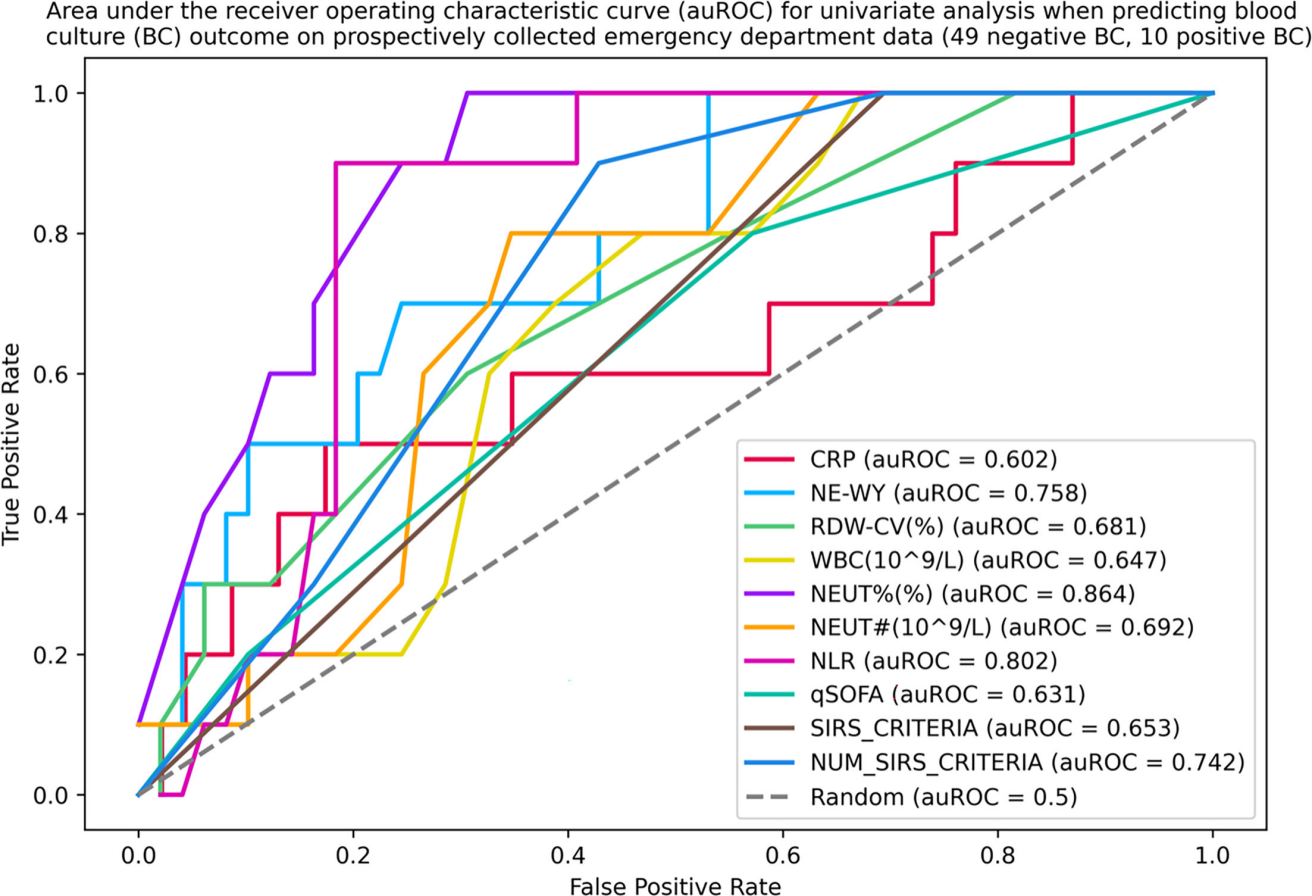
auROC for univariate cut-off classification for prediction of BC outcomes on the entire dataset (49 negative BCs, 10 positive BCs). The variables evaluated include C-reactive protein (CRP), width of dispersion of neutrophils fluorescence (NE-WY), red blood cell distribution width (RDW-CV(%)), white blood cell count (WBC(10^9^/L)), neutrophil differential relative percentage (NEUT%(%)), absolute neutrophil count (NEUT#(10^9^/L)), neutrophil–lymphocyte ratio (NLR), quick sequential organ failure assessment (qSOFA) score, patient meets the systemic inflammatory response syndrome (SIRS) criteria (SIRS_CRITERIA) and number of SIRS clinical elements that the patient meets (NUM_SIRS_CRITERIA).

In addition to the multivariate models, we also explored the predictive potential of individual laboratory variables through univariate cut-off analysis, where a positive (POS) prediction was made if the value of a specific variable exceeded a predefined threshold. The ROC curves and auROC values for CRP, neutrophil fluorescence width of dispersion (NE-WY), RBC distribution width (RDW-CV [%]), EBC count (WBC [10⁹/L]), neutrophil differential percentage (NEUT% [%]), absolute neutrophil count (NEUT# [10⁹/L]) and NLR are shown in Fig. 3.

When evaluated using bootstrap resampling, NEUT%, NLR and NE-WY emerged as the strongest univariate predictors, achieving mean auROC scores of 0.851 (95% CI, 0.753–0.931), 0.792 (95% CI, 0.686–0.901) and 0.745 (95% CI, 0.590–0.887), respectively. In contrast, CRP displayed a comparatively weaker discriminative performance, with a mean auROC of 0.589 (95% CI, 0.359–0.799).

The univariate analysis was also performed with the qSOFA and SIRS criteria. We evaluated the performance of the following for BC outcome classification: (1) Patient meets the qSOFA criteria, (2) patient meets the SIRS criteria and (3) the number of SIRS clinical elements that the patient meets. The mean auROC for each of these approaches was 0.630 (95% CI, 0.455–0.800) for qSOFA criteria, 0.653 (95% CI, 0.589–0.729) for SIRS criteria and 0.738 (95% CI, 0.600–0.849) for the number of SIRS clinical elements met.

These results highlight that while certain haematological parameters and clinical scoring systems display moderate predictive value, ensemble learning models such as RF and XG improve overall classification performance. All results have been reported for uncalibrated models, as these were evaluated in our previous work. We have included the calibration plots for both the calibrated and uncalibrated models in Figs S5–S8.

## Discussion

**Fig. 4.**
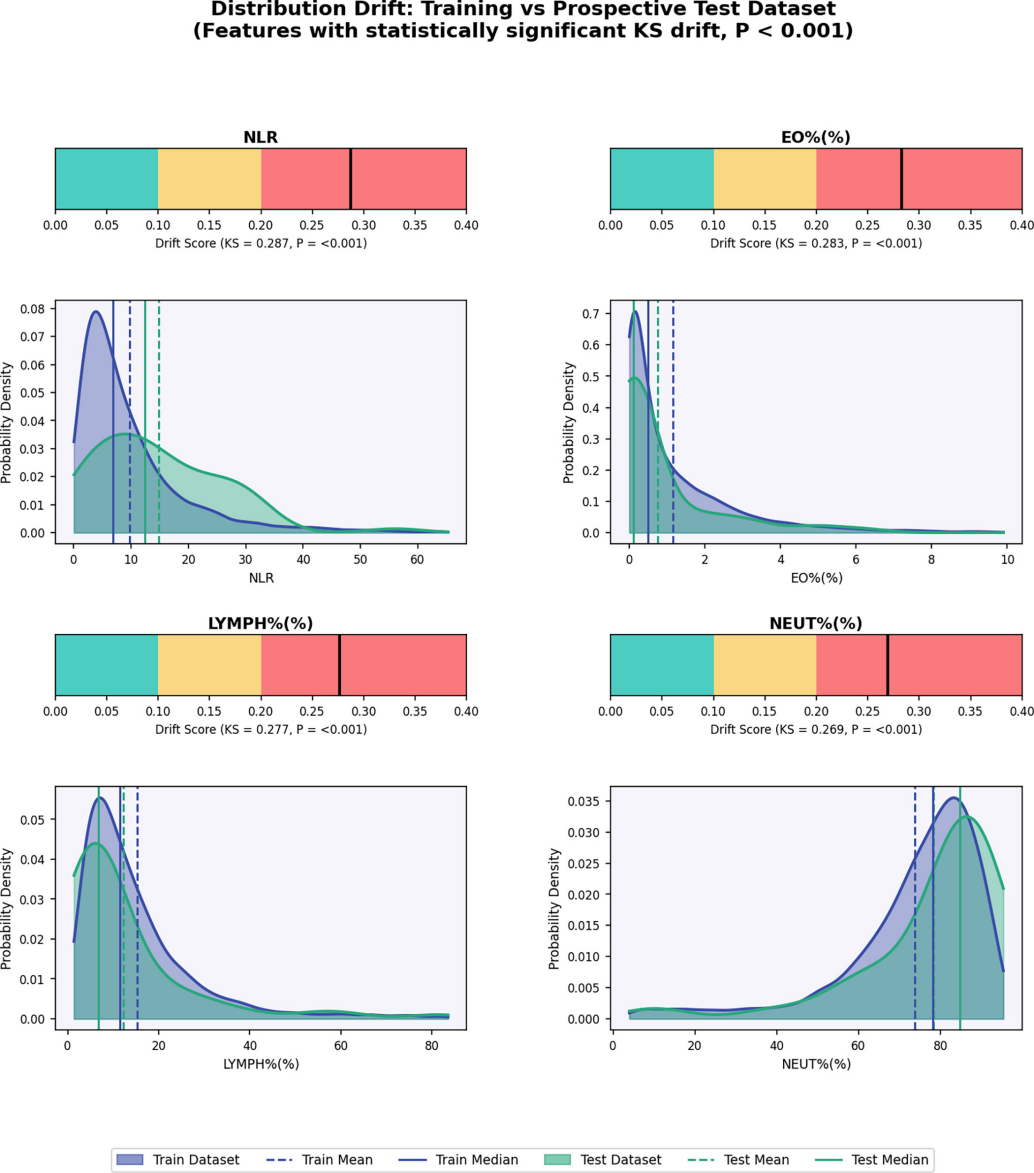
Data drift for NLR, neutrophil percentage (NEUT%(%)), lymphocyte percentage (LYMPH%(%)) and eosinophil percentage (EO%(%)) between the original training dataset (Train) and the ED dataset presented in this study (Test). These features are displayed due to having statistically significant Kolmogorov–Smirnov (KS) test drift.

In this study, we evaluated previously trained ML models for BC outcome prediction using a prospectively collected ED dataset. Despite being trained with heterogeneous patient population samples, both the RF and XG models were capable of generalizing to the ED dataset, achieving high performance. We also evaluated the performance of univariate cut-off classification to compare with the results produced by the ML models. The RF model, XG model, NEUT%(%) and NLR all performed similarly when evaluated on the entire dataset with auROC scores of 0.861, 0.835, 0.864 and 0.802 respectively. These all outperformed CRP, which had an auROC of 0.602 and qSOFA, SIRS and the number of SIRS clinical elements with auROC scores of 0.631, 0.653 and 0.742, respectively. However, when evaluating performance via bootstrapping, the RF model achieved the highest mean auROC followed by NEUT%(%) and the XG model. This is presented in Table 3. Interestingly, the high performance of the ML models and high auROC scores of NEUT%(%) and NLR can largely be explained by the differences in distribution of the features in the ED dataset when compared with the previously used heterogeneous training dataset. This is shown by the feature drift between the original training dataset and the dataset presented in this study. We demonstrate the drift with the deepchecks Python library [27], using the Kolmogorov–Smirnov test. Of particular interest is the feature drift detected for NLR, NEUT%(%), LYMPH%(%) and EO%(%) which is shown in Fig. 4. Distributional differences for all features are shown in Table 4.

**Table 3.** auROC scores for different methods in predicting positive BC outcome. Results are presented as mean auROC (95% confidence interval, lower bound–upper bound) as a result of 1,000 bootstrap replications. NEUT%(%) (neutrophil differential relative percentage); NLR (neutrophil–lymphocyte ratio); CRP (C-reactive protein); quick sequential organ failure assessment (qSOFA) score; patient meets the systemic inflammatory response syndrome (SIRS) criteria (SIRS_CRITERIA); number of SIRS clinical elements that the patient meets (NUM_SIRS_CRITERIA)

Method	Mean auROC (95% confidence interval, lower bound–upper bound)
XG	0.833 (95% CI, 0.683–0.953)
RF	0.865 (95% CI, 0.763–0.947)
NEUT%(%)	0.851 (95% CI, 0.753–0.931)
NLR	0.792 (95% CI, 0.686–0.901)
CRP	0.589 (95% CI, 0.359–0.799)
qSOFA	0.630 (95% CI, 0.455–0.800)
SIRS_CRITERIA	0.653 (95% CI, 0.589–0.729)
NUM_SIRS_CRITERIA	0.738 (95% CI, 0.600–0.849)

**Table 4.** Distributional differences for features were assessed using the Kolmogorov–Smirnov (KS) test for continuous variables. The KS statistic indicates the maximum difference in probability distribution shape (where 0 indicates identical distributions and values>0.2 suggest substantial drift). Cohen’s *d* was calculated as a standardized effect size for continuous variables, with the sign indicating the direction of shift (positive: higher mean in the training set; negative: higher mean in the test set) and the magnitude reflecting the degree of difference (~0.2=small, ~0.5=medium, ~0.8=large). A significance threshold of *P*<0.05 was applied. Neutrophil fluorescence intensity ([NE-SFL(ch)]); neutrophils forward scatter ([NE-FSC(ch)]); width of dispersion of neutrophils fluorescence ([NE-WY]); width of dispersion of monocytes fluorescence ([MO-WY]); width of dispersion of monocytes size ([MO-WZ]); red blood cell distribution width (RDW-CV(%)); platelet count (PLT(10^9^/L)); haemoglobin (HGB(g/L)); red blood cell count (RBC(10^12^/L)); white blood cell count (WBC(10^9^/L)); monocyte differential relative percentage (MONO%(%)); basophil differential relative percentage (BASO%(%)); eosinophil differential relative percentage (EO%(%)); lymphocyte differential relative percentage (LYMPH%(%)); neutrophil differential relative percentage (NEUT%(%)); absolute basophil count (BASO#(109 /L)); absolute monocyte count (MONO#(10^9^ /L)); absolute eosinophil count (EO#(10^9^ /L)); absolute lymphocyte count (LYMPH#(10^9^/L)); absolute neutrophil count (NEUT#(10^9^/L)); neutrophil–lymphocyte ratio (NLR); monocyte–lymphocyte ratio (MLR)

Feature	Type	KS statistic	P-Value	Effect size
[NE-SFL(ch)]	Numerical	0.117	0.368	0.153
[NE-FSC(ch)]	Numerical	0.132	0.237	−0.068
[NE-WY]	Numerical	0.169	0.061	−0.04
[MO-WY]	Numerical	0.156	0.101	−0.233
[MO-WZ]	Numerical	0.109	0.456	−0.232
RDW-CV(%)	Numerical	0.158	0.094	0.279
PLT(10^9^ /L)	Numerical	0.111	0.426	0.161
HGB(g/L)	Numerical	0.232	0.003	−0.347
RBC(10^12^ /L)	Numerical	0.175	0.047	−0.236
WBC(10^9^ /L)	Numerical	0.177	0.044	−0.097
MONO%(%)	Numerical	0.121	0.328	0.098
BASO%(%)	Numerical	0.199	0.016	0.192
EO%(%)	Numerical	0.283	<0.001	0.238
LYMPH%(%)	Numerical	0.277	<0.001	0.241
NEUT%(%)	Numerical	0.269	<0.001	−0.274
BASO#(10^9^ /L)	Numerical	0.093	0.659	0.054
MONO#(10^9^ /L)	Numerical	0.151	0.121	0.021
EO#(10^9^ /L)	Numerical	0.237	0.002	0.191
LYMPH#(10^9^ /L)	Numerical	0.204	0.013	0.096
NEUT#(10^9^ /L)	Numerical	0.218	0.006	−0.252
NLR	Numerical	0.287	<0.001	−0.238
MLR	Numerical	0.224	0.005	−0.248

The research presented in this article contributes to the growing body of literature surrounding the development of ML models for bacteraemia and BC outcome prediction in EDs. Chang *et al.* developed ML models using CBC, DIFF and CPD features produced by the Beckman Coulter DxH900 analyser. Their CatBoost model achieved an auROC of 0.844 and 0.847 in two external validation datasets [28]. In their study, BC samples were obtained within 4 h before or after collection of blood test samples. Boerman *et al.* developed ML models for identifying patients at risk of bacteraemia [29]. They developed a gradient boosted tree model and a logistic regression model, with auROC scores of 0.770 and 0.780, respectively, for the test sets. The models were developed using demographic information, vital signs, laboratory results, radiology results and medications administered in the ED. Schinkel *et al.* developed an XG model for BC outcome prediction that obtained auROC scores of 0.800, 0.760 and 0.750 across three different validation cohorts. The model also achieved an auROC of 0.760 in prospective evaluation. The model was trained using laboratory data, vital signs and patient demographics [30]. Tsai *et al.* developed ML models for prediction of bacteraemia for adult febrile ED patients. RF and logistic regression models achieved 0.761 and 0.755 auROC scores on the derivation datasets. Their RF model obtained 0.709 in the validation dataset [31]. Choi *et al*. developed ML models for prediction of bacteraemia at the ED during triage and disposition. A triage XG model obtained an auROC of 0.718 and the disposition XG model obtained an auROC of 0.853, both for the validation datasets [32]. Our approach of performing the original model training on a heterogeneous population and subsequently obtaining high performance on a more specific patient population has been previously discussed by Schinkel *et al.* [33]. Recently, a randomized, controlled, non-inferiority trial that compares standard practice with an ML-guided approach to BC outcome prediction has been proposed [34], with the aim to determine whether the ML-based method is non-inferior to current practice in terms of 30-day mortality. Similar approaches used for BC outcome prediction have also been applied elsewhere. Lin *et al*. [35] applied ML methods to CBC and DIFF data for the purpose of predicting sepsis in patients admitted through the ED. Their light gradient boosting machine model achieved an auROC of 0.90. They integrated their model into a web application as part of their artificial intelligence clinical decision support system. Ming *et al.* [36] compared the use of a long short-term memory (LSTM) model with time series data and a static logistic regression model for the prediction of BC results. In the hold-out test set, the static model obtained an auROC of 0.74 (95% CI, 0.70–0.78), and the LSTM obtained an auROC of 0.97 (95% CI, 0.96–0.97). They highlighted the importance of time series information for improving performance. Their study focused on patients admitted to and staying in hospital. Another study focusing on hospitalized patients was performed by Murri *et al.* [37]. They developed a logistic regression model to discriminate between those at low and high risk of BSI. Their model obtained an auROC of 0.74.

Given the increasing body of literature and consistent performance across different studies, future work should aim to focus on how to deploy bacteraemia and BC outcome prediction models in practice. Two possible options are (1) a process that suspends further BC testing in the case of a negative prediction, as proposed in our previous study, or (2) applying a modification to the existing laboratory process, and rather than stop BCs being performed, a two-stream approach is implemented which aims to provide a ‘fast-track’ alternative for subsequent rapid pathogen identification and antimicrobial susceptibility testing. In this case, samples that are predicted to be positive would go down the ‘fast-track’ pathway, and negative predictions would remain on the standard BC pathway. Option 1 is higher risk and focuses on improving diagnostic stewardship and resource expenditure. Option 2 is lower risk, as it does not completely change the standard clinical workflow but increases the speed at which detection of BSIs and antimicrobial susceptibility testing is performed for higher risk patients. We have also made the code that was developed for model training and evaluation available as open-source at the GitHub repository https://github.com/benjaminmcf/blood-culture-outcome-classification/. The code can be used by others to replicate the training and evaluation process highlighted in this article and our previous work [19]. The code can be extended to integrate models into web applications or application programming interfaces to support further studies/trials. The code in the repository is written in the Python programming language (version 3.12), with further instructions regarding the use of the code available in the repository.

This study has several limitations that need to be acknowledged. The first is the relatively small size of the validation dataset. Due to the prospective, time-limited nature of this study, additional data collection was not possible. Given this, future work should aim to repeat the analysis across a larger ED dataset to further validate the models. Second, only patients that had CBC blood samples and BC samples collected at the same time were included. Therefore, this study excludes patients who had BCs and CBC samples taken at different times. Supporting this capability would require retraining the model to be used for patients that had CBC and BC samples collected at different times. Third, there is also possible selection bias due to limiting patients to those who were able to provide consent. Therefore, the dataset excludes those patients that either (1) did not want to provide consent and (2) those that were too unwell to provide consent. Furthermore, samples were only included if clinicians requested a BC for the patient. Therefore, the model relies on an initial clinical judgement by the ED clinical staff. Fourth, in line with the original development of the model, additional clinical information about patients including comorbidities and symptoms on presentation to the ED was not included in the model or during the predictive process. Whilst this was a deliberate choice, it is worth noting as a potential limitation. When developing and evaluating our ML models, we used the BC results as they are routinely reported in the laboratory as our ground truth for BSI. In future work, it would be beneficial to explore alternative approaches such as nucleic acid amplification techniques or other culture-free bacterial detection methods for the purposes of establishing the ground truth. We used data obtained from the Sysmex XN-2000 analysers, as these are the haematology analysers used in Western Australia where the blood samples were processed. The models could be applied to other haematology analysers such as the Beckman Coulter DxH900 analyser (Beckman Coulter, Miami, Florida, USA), as the two systems have demonstrated strongly correlated results [38]. However, this would have to be validated in further analysis. Lastly, because ethics approval was granted for a single site, the study was limited to a single ED in Western Australia, with further validation in multiple EDs required to assess the wider applicability of these models.

## Supplementary material

10.1099/jmm.0.002191 Supplementary Material 1.

## Abbreviations

auROC: area under the receiver operating characteristic curve
BC: blood culture
BFS: boruta feature selection
BSI: bloodstream infection
CBC: complete blood count
CI: confidence interval
CPD: cell population data
CRP: C-reactive protein
DIFF: white blood cell differential
ED: emergency department
FSC: forward scatter light
IQR: interquartile range
LIS: laboratory information system
LSTM: long short-term memory
ML: machine learning
MLR: monocyte–lymphocyte ratio
NLR: neutrophil–lymphocyte ratio
NPV: negative predictive value
PPV: positive predictive value
qSOFA: quick sequential organ failure assessment
RF: random forest
SCGH: Sir Charles Gairdner Hospital
SFL: fluorescent light intensity
SIRS: systemic inflammatory response syndrome
SSC: side scatter light
XG: XGBoost
