# Supplementary material for: Evaluation of machine learning pipeline for blood culture outcome prediction on prospectively collected emergency department data
Source: J Med Microbiol. 2026 Jul 29;75(7):002191. doi: 10.1099/jmm.0.002191 (PMC13419127; doi:10.1099/jmm.0.002191)
Supplement: Supplementary Material 1. [file jmm-75-02191-s001.pdf]

Supplemental Figure 1: Confusion matrices for the uncalibrated random forest (RF) model at classification thresholds 0.1 - 0.9

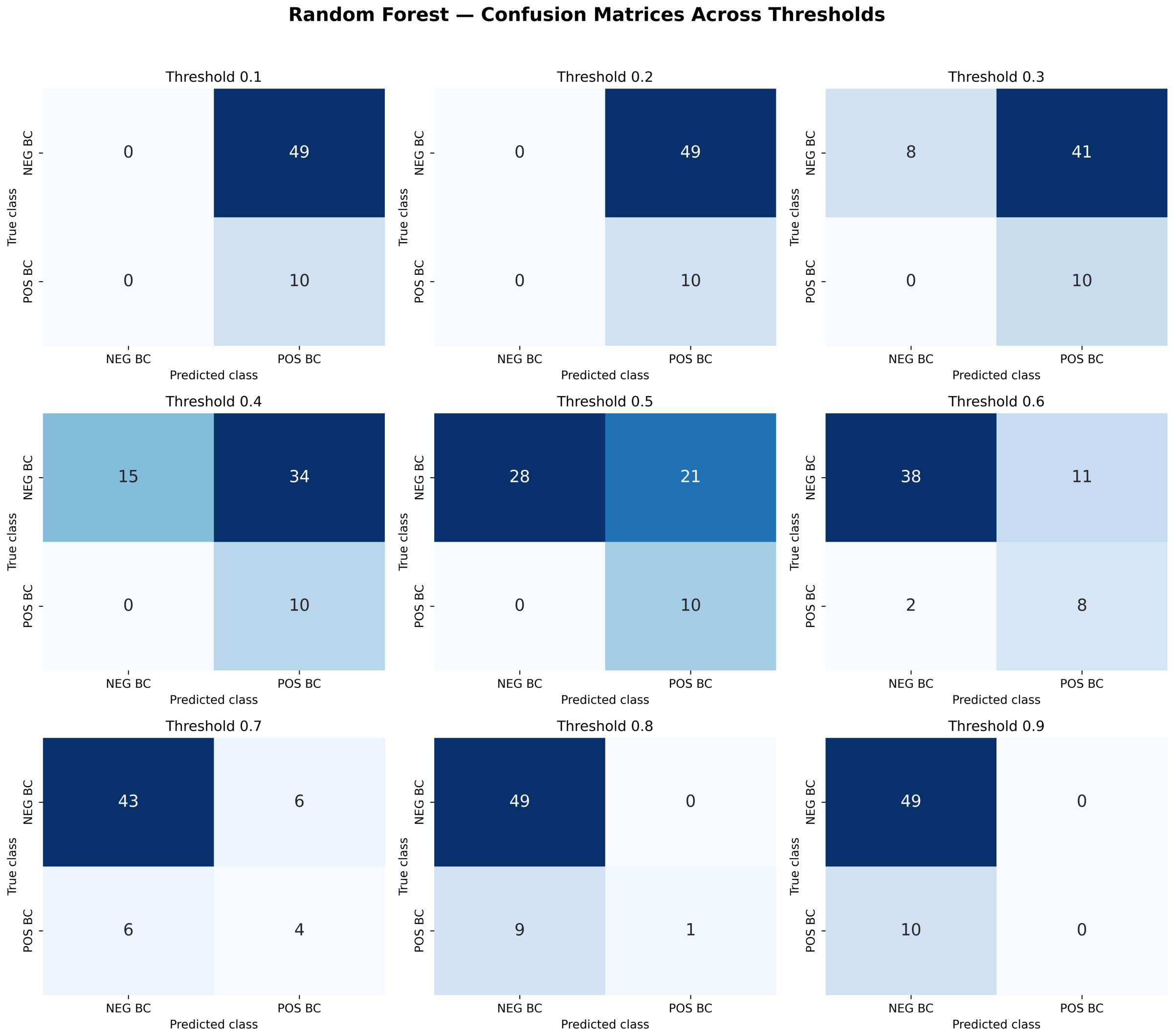

Supplemental Figure 2: Confusion matrices for the calibrated random forest (RF) model at classification thresholds 0.1 - 0.9

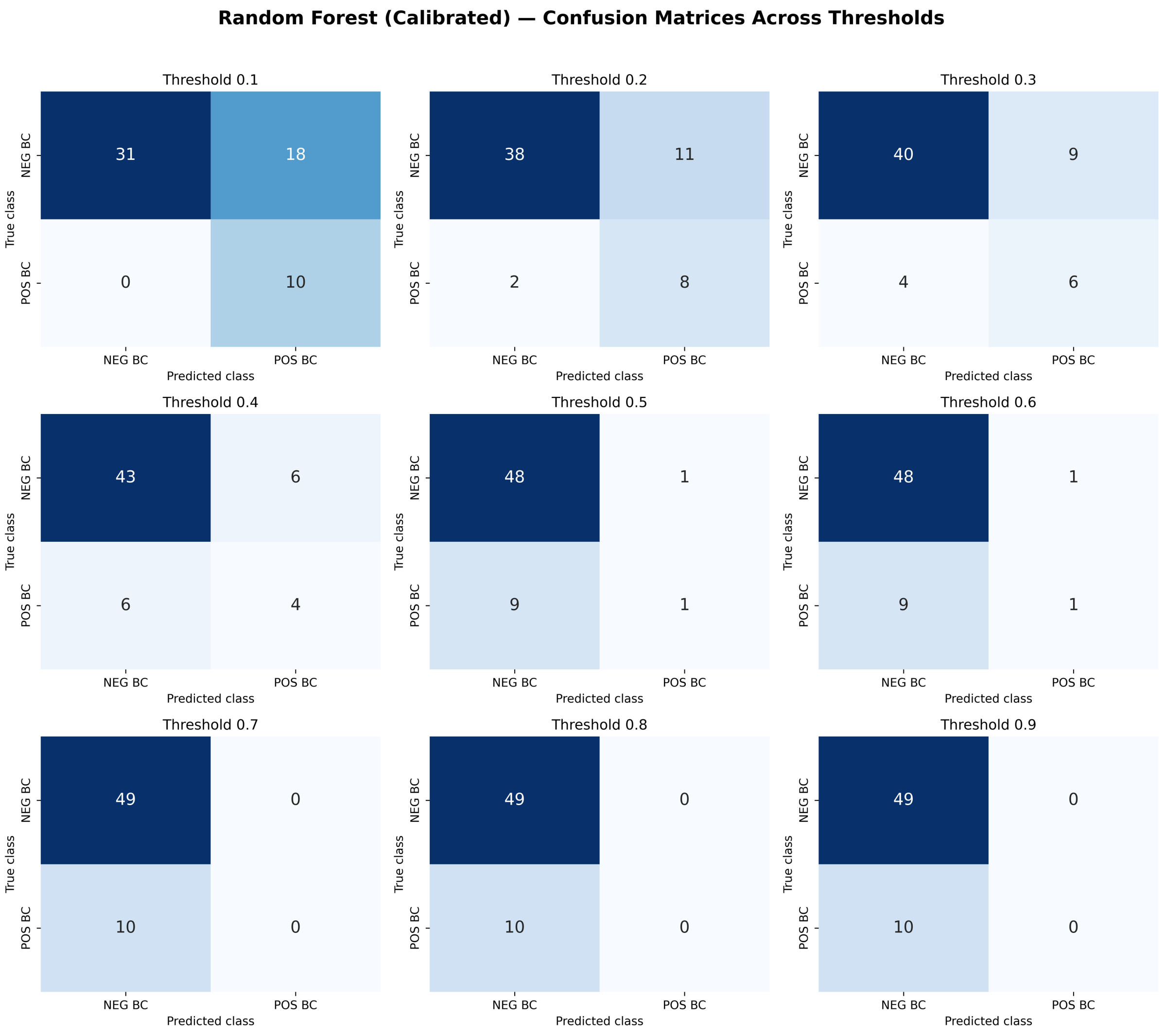

Supplemental Figure 3: Confusion matrices for the uncalibrated XGBoost (XG) model at classification thresholds 0.1 - 0.9

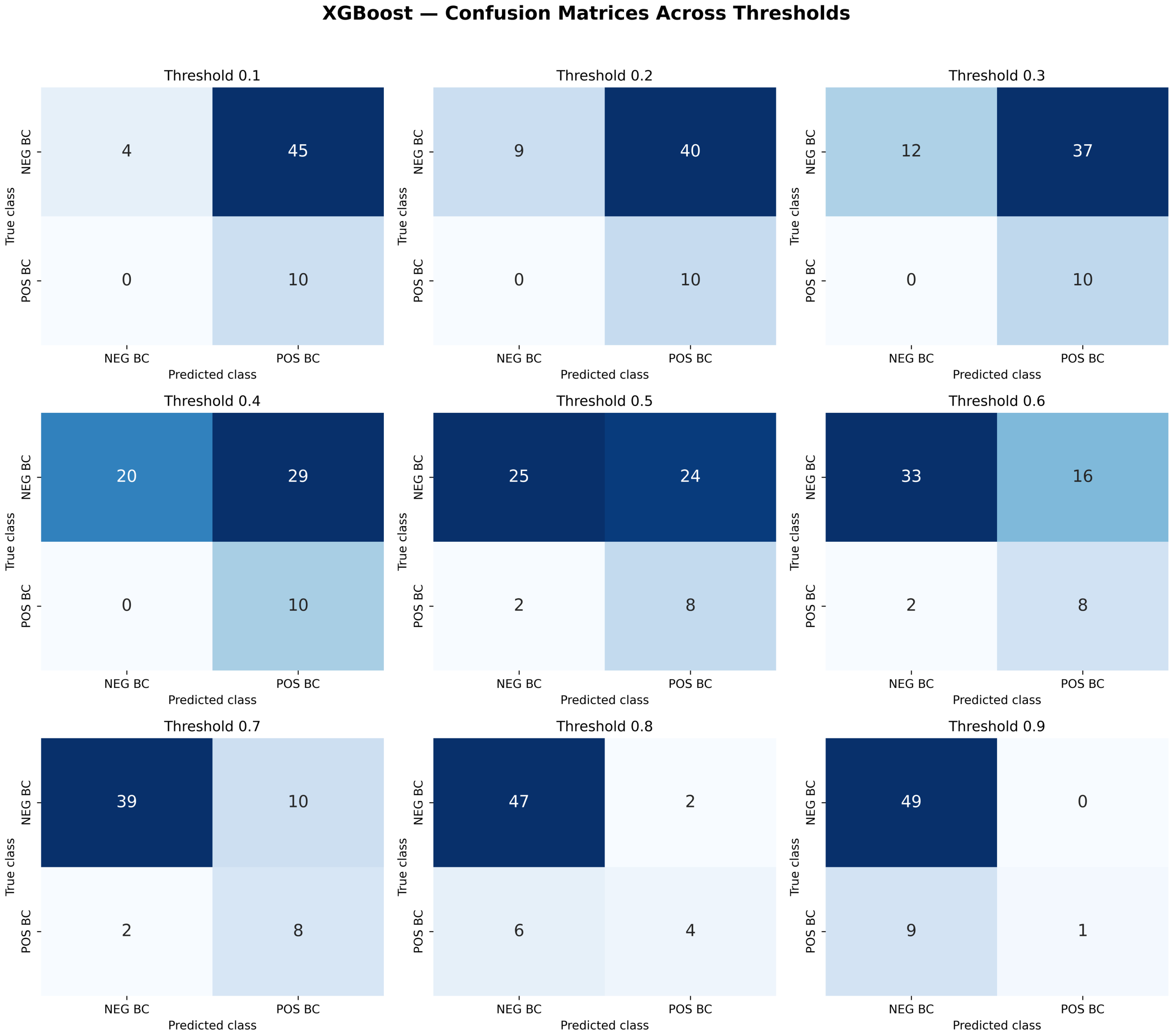

Supplemental Figure 4: Confusion matrices for the calibrated XGBoost (XG) model at classification thresholds 0.1 - 0.9

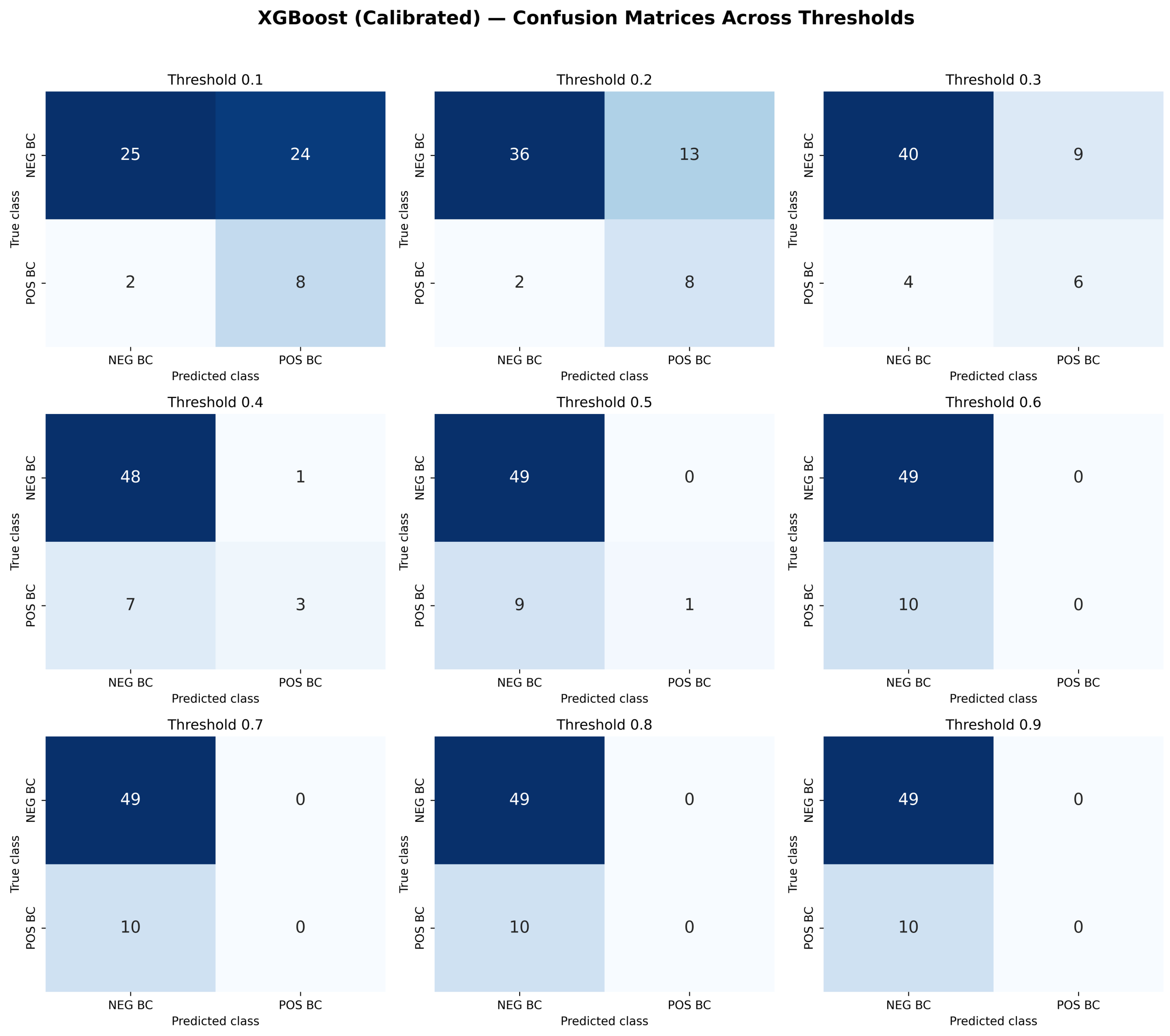

Supplemental Figure 5: Calibration curve for the uncalibrated random forest (RF) model

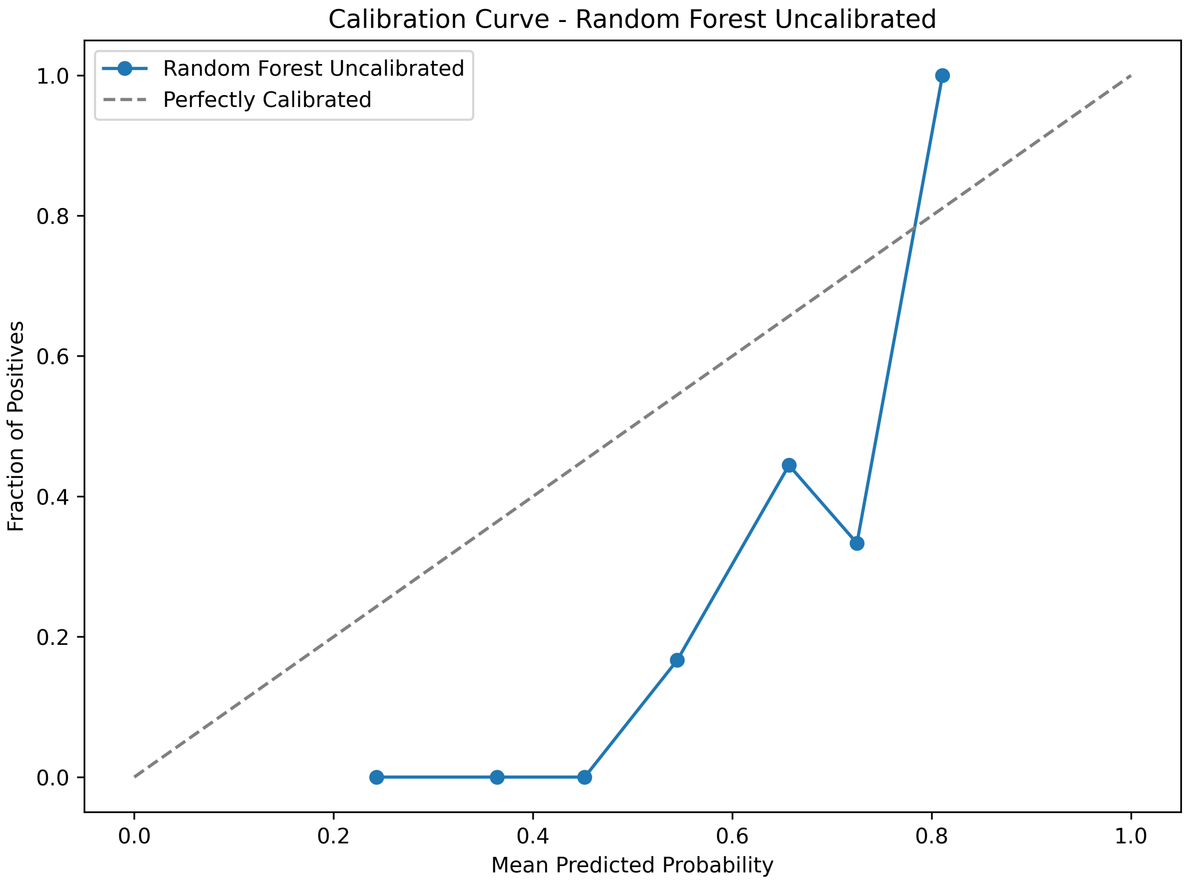

Supplemental Figure 6: Calibration curve for the calibrated random forest (RF) model

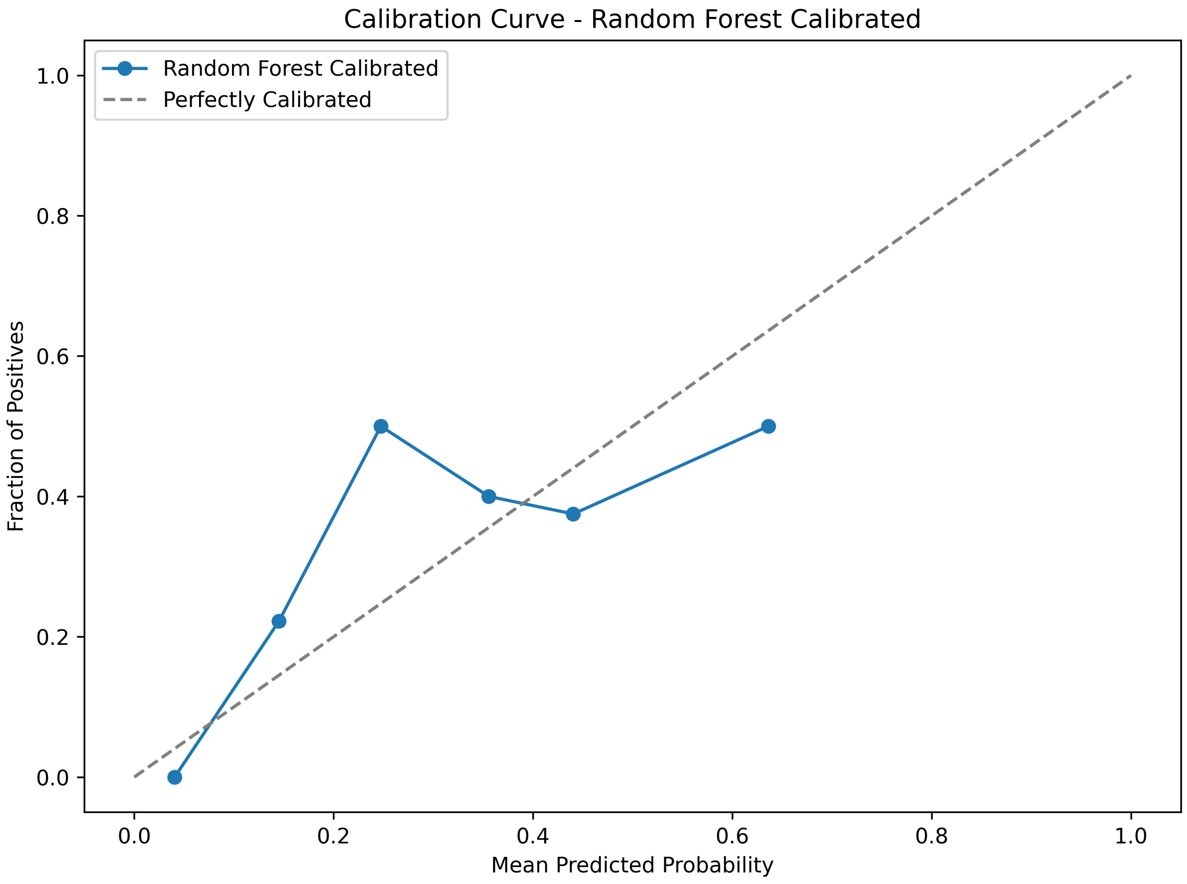

Supplemental Figure 7: Calibration curve for the uncalibrated XGBoost (XG) model

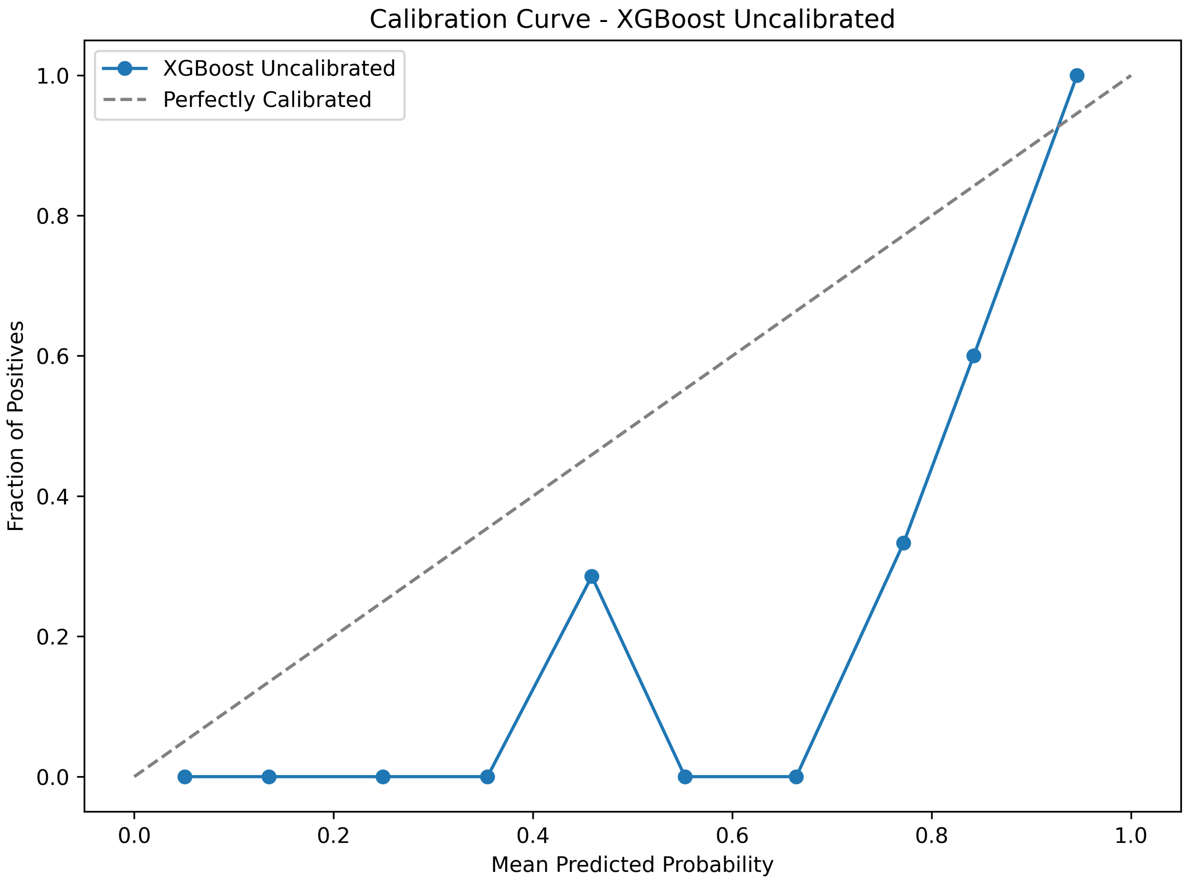

Supplemental Figure 8: Calibration curve for the calibrated XGBoost (XG) model

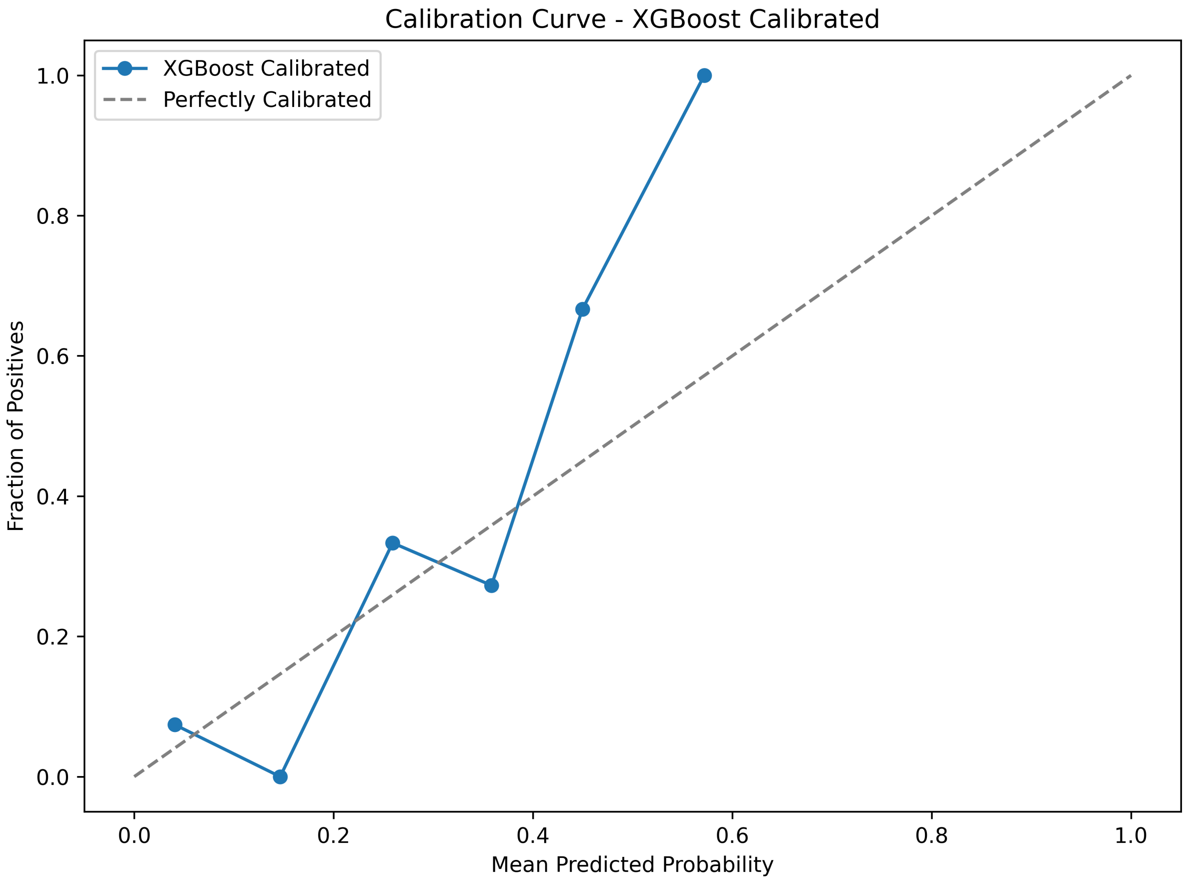

Supplemental Figure 9: Equations for neutrophil-lymphocyte ratio and monocyte-lymphocyte ratio

$$Neutrophil - to - lymphocyte\ ratio\ (NLR) = \frac{NEUT\ count}{LYMPH\ count}$$

$$Monocyte - to - \ lymphocyte\ ratio\ (MLR) = \frac{MONO\ count}{LYMPH\ count}$$

Supplemental Table 1: Table showing the different metrics used to evaluate the machine learning models

Metrics	Description	Equation
True positives (TP)	Number of instances that the model correctly classifies as positive.	-
False negatives (FN)	Number of instances that are positive but are incorrectly classified as negative by the model.	-
True negatives (TN)	Number of negative class instances that the model correctly identifies as negative.	-
False positives (FP)	Number of instances that are negative but are incorrectly classified as positive by the model.	-
Sensitivity	Sensitivity, also known as True Positive Rate (TPR) or Recall, is a metric that measures the ability for a machine learning model to correctly identify positive class instances from all the true positive instances in a dataset. Sensitivity is particularly important when solving binary classification problems,	$Sensitivity(Recall) = \frac{TP}{TP + FN}$

	where the goal is to correctly distinguish between two classes, often labelled as "positive" and “negative". Sensitivity measures the model's ability to identify all the relevant positive class instances, and it is crucial in scenarios where missing positive instances has high consequences, such as in medical diagnosis, or in any domain where the cost of false negatives is high. A high sensitivity indicates that the model is good at identifying positive cases instances, but it may come at the expense of higher false positives.	
Specificity	Specificity refers to the ability of a machine learning model to correctly identify negative class instances. It is a metric that is used to determine how effectively a model is at avoiding false positive predictions. It can also be defined as the ability for a model to correctly identify the negative class instances. High specificity scores indicate that the model can avoid false positive predictions, where depending on the context that the model is being used, can be critical.	$Specificity = \frac{TN}{TN + FP}$
auROC	The auROC refers to the area under the receiver operating characteristic (ROC) curve. The ROC is a plot of the true positive rate (sensitivity) against false positive rate (1-	-

	specificity) at various classification thresholds. The AUC provides a summation of the performance across all the various classification thresholds. An AUC of 1 indicated that the model can perfectly distinguish between the positive and negative classes. An AUC of 0.5 implies that the model is no better than random guessing. Any model that achieves an AUC less than 0.5 is therefore worse than random guessing.	
Positive predictive value (PPV)	The PPV is a metric that refers to the proportion of positive class predictions made by the model that were positive. It is determined by the number of TP divided by the total number of instances that are predicted as positive (TP + FP).	$PPV = \frac{TP}{TP + FP}$
Negative predictive value (NPV)	The NPV is a metric that refers to the proportion of negative class predictions made by the model that were negative. It is determined by the number of TN divided by the total number of instances that are predicted as negative (TN + FN)	$NPV = \frac{TN}{TN + FN}$
